# Ischemic dermal pallor as an unusual cutaneous clue for group A streptococcal necrotizing soft tissue infection

**DOI:** 10.1016/j.jdcr.2026.05.040

**Published:** 2026-05-25

**Authors:** Tiffany Leng, Nicole Grbic, Martin Azzam, Candice Brem, Allison Perz, Alejandro Barrera-Godínez

**Affiliations:** a Boston University Aram V. Chobanian & Edward Avedisian School of Medicine, Boston, Massachusetts; b Department of Dermatology, Boston Medical Center, Boston, Massachusetts

**Keywords:** eschar, GAS NSTI, group A Strep, ischemic pallor, necrotizing soft tissue infection, occlusive vasculopathy

## Introduction

Group A *Streptococcus* (GAS) is one of the leading causes of necrotizing soft tissue infections (NSTIs), with a case-fatality rate as high as 29% in the U.S.^1^ The presentation of NSTIs can vary widely, and intervention should be started as soon as possible once clinical suspicion is raised. Key early classically described diagnostic clues include disproportionate pain, edema beyond erythema, small vesicles, crepitus, skin bullae, or ecchymosis.^2^^,^^3^ Severe advanced stage signs include hemorrhagic bullae, crepitus, hypoesthesia, gangrene, and septic shock.^2^^,^^3^ However, early cutaneous findings may be subtle or atypical, contributing to delays in diagnosis. We describe a striking porcelain-white appearance of exposed dermis as an early manifestation of NSTI, not typically emphasized in prior literature. Recognition of this atypical presentation may facilitate earlier diagnosis in cases lacking classic signs.

## Case report

We present a case of GAS NSTI with a more uncommon clinical appearance. A 41-year-old woman presented with rapidly progressive, superficial, bullous-macerated skin on the pannus, along with fever, tachycardia, and hypotension. She first experienced painful abdominal blisters, fevers, and chills 2 days prior while on vacation in Puerto Rico, where she also swam and consumed raw seafood. New-onset nausea, vomiting, diarrhea, and dry cough prompted her to present to the emergency department upon returning home. Her cutaneous findings progressed rapidly over approximately 48 hours. Her past medical history was notable for heart failure with preserved ejection fraction, iron-deficiency anemia that is managed with iron infusions, and type 2 diabetes. Additionally, she underwent a total abdominal hysterectomy with bilateral salpingo-oophorectomy 3 weeks earlier for endometrioid intraepithelial neoplasia.

**Fig 1:**
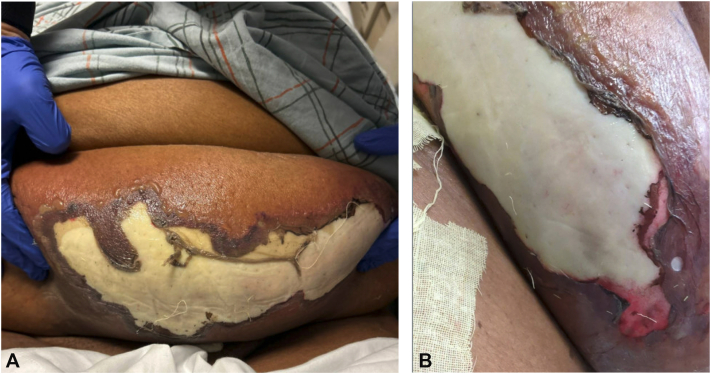
**A** and **B,** Porcelain-white exposed dermis and surrounding purpuric retiform margins on the lower abdomen.

Examination revealed along the lower abdomen a large ulceration with exposed dermis exhibiting a porcelain-white, avascular appearance, and surrounding purpuric retiform margins (Fig 1). She was empirically started on doxycycline, cefepime, and vancomycin after meeting sepsis criteria in the setting of a possible skin and soft tissue infection. The initial differential diagnosis included NSTI, ecthyma gangrenosum, *Vibrio* infection given the recent marine exposure and raw seafood ingestion, and disseminated intravascular coagulation from sepsis.

**Fig 2:**
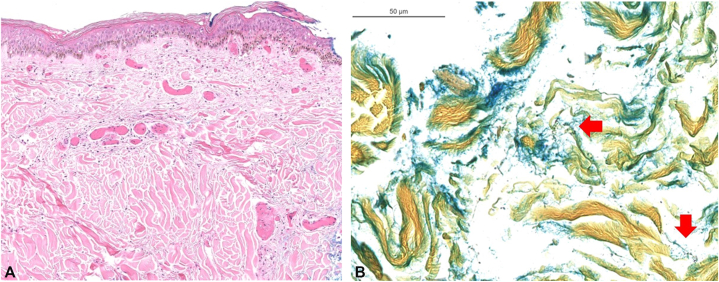
**A,** Histology section from punch biopsy showing dilated and congested small-caliber blood vessels with occasional intraluminal fibrin thrombi in the superficial and mid dermis, and a sparse-to-mild superficial and mid perivascular lymphocytic infiltrate with occasional neutrophils. (H&E, 10×). **B,** Gram stain highlighted rare Gram (+) cocci (*red arrows*) in the superficial and deep dermis. (Gram, 40×).

Dermatology was consulted approximately 24 hours after admission. A punch biopsy was obtained from the ulcer's edge, which exhibited superficial epidermal necrosis, fibrin thrombi within congested dermal vessels, and a sparse inflammatory infiltrate (Fig 2, *A*). Subsequent tissue Gram stain demonstrated intermixed Gram-positive cocci in the dermis (Fig 2, *B*) and tissue culture yielded beta-hemolytic group A *Streptococcus* (*S. pyogenes*). Three days after admission, the patient was transitioned from empiric antibiotics to cefazolin and linezolid for antitoxin treatment, surgical debridement of necrotic tissue was performed, and she was eventually discharged with a wound VAC.

## Discussion

A striking feature in this patient’s presentation was the porcelain-white appearance of the exposed dermis, likely reflecting early ischemic injury prior to overt necrotic discoloration, consistent with histopathological evidence of pauci-inflammatory cutaneous vascular occlusion. The dermal pallor was likely mediated by GAS exotoxins that promote platelet-leukocyte complex formation, resulting in vascular occlusion and reduced dermal perfusion. Specifically, streptolysin O has been shown to lead to vascular occlusion through integrin interactions with platelets and leukocytes.^4^ However, ischemia due to platelet-leukocyte complexes is not unique to GAS NSTIs and has been reported in *Clostridium perfringens* infections via different mechanisms.^5^

Most GAS NSTIs commonly feature prominent dermal edema and polymorphonuclear infiltration.^6^ However, this patient’s biopsy revealed only a sparse infiltrate, an unexpectedly muted response given the severity of infection. This paradoxical lack of inflammation was likely driven by anti-inflammatory GAS virulence factors. Peptidases ScpA and SpyCEP can degrade chemotactic C5a peptide and interleukin-8, inhibiting neutrophil recruitment.^7^ Similarly, streptococcal pyrogenic exotoxin B can cleave other proinflammatory factors.^8^ The streptolysin O-mediated platelet-leukocyte complexes previously described also impair neutrophil diapedesis via endothelial occlusion.^4^^,^^5^ Studies have suggested that a lack of polymorphonuclear leukocytes, indicating minimal to absent neutrophilic response, correlates with more advanced disease and higher mortality in NSTI.^9^ Despite this histologic finding, our patient’s clinical condition did not match typical advanced-stage GAS NSTI. The timing of the skin biopsy, obtained 24 hours after antibiotics, may have further contributed to the muted inflammatory response.

A porcelain-white appearance of exposed dermis as an early manifestation of NSTI has not, to our knowledge, been explicitly emphasized in prior literature.^10^ In this case, local friction from clothing might have contributed to early detachment of the necrotic epidermis, allowing visualization of the ischemic dermis prior to eschar formation. We believe porcelain-white dermal pallor may represent an underrecognized early manifestation of NSTI. The differential diagnosis could include early stages of other vaso-occlusive processes, such as ecthyma gangrenosum, calciphylaxis, and disseminated intravascular coagulation, which may be distinguished by the clinical context and histopathological findings.

The discordant constellation of occlusive vasculopathy and minimal tissue inflammation in the context of an NSTI underscores the complex interplay of GAS virulence factors. This pathophysiology may manifest as a distinct cutaneous finding of porcelain-white dermis, which should prompt clinicians to consider NSTI when recognized, even in the absence of classic signs of inflammation. Early identification of these atypical findings enables prompt diagnosis and treatment, reducing reliance on confirmatory cultures and potentially fatal delays.

## Conflicts of interest

None disclosed.
